# Pemphigus Vulgaris Presented with Cheilitis

**DOI:** 10.1155/2014/147197

**Published:** 2014-09-25

**Authors:** Zaheer Abbas, Zahra Safaie Naraghi, Elham Behrangi

**Affiliations:** ^1^Department of Dermatology, Razi Hospital, Tehran University of Medical Sciences, Vahdate Eslami Square, Vahdate Eslami Avenue, Tehran 11996, Iran; ^2^Department of Dermatology, Rasoul-e Akram Hospital, Iran University of Medical Sciences, Tehran, Iran

## Abstract

*Background*. Pemphigus vulgaris is an autoimmune blistering disease affecting the mucous membrane and skin. In 50 to 70% of cases, the initial manifestations of pemphigus vulgaris are oral lesions which may be followed by skin lesions. But it is unusual for the disease to present with initial and solitary persistent lower lip lesions without progression to any other location. *Main Observations*. We report a 41-year-old woman with dry crusted lesions only on the lower lip, clinically resembling actinic cheilitis and erosive lichen planus, but histopathological evaluation showed unexpected results of suprabasal acantholysis and cleft compatible with pemphigus vulgaris. We treated her with intralesional triamcinolone 10 mg/mL for 2 sessions and 2 g cellcept daily. Patient showed excellent response and lesions resolved completely within 2 months. In one-year follow-up, there was no evidence of relapse or any additional lesion on the other sites. *Conclusion*. Cheilitis may be the initial and sole manifestation of pemphigus vulgaris. Localized and solitary lesions of pemphigus vulgaris can be treated and controlled without systemic corticosteroids.

## 1. Introduction

Pemphigus vulgaris (PV) is an autoimmune intraepithelial blistering disease involving mucous membranes and the skin. The oral mucous membrane is frequently affected in PV patients; most of patients present with oral lesions as the first sign of PV [1, 2]. Lesions may occur anywhere on the oral mucosa, but the buccal mucosa is the most commonly affected site, followed by involvement of the palatal, lingual, labial mucosae, and the gingiva [3]. Here we present a case of PV manifested as persistent crusted lesions only on the lower lip.

## 2. Case Report

**Figure 1:**
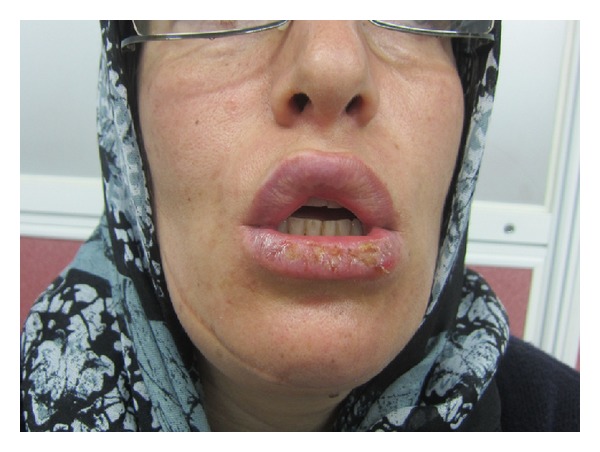
Scaly crusted lesions on lower lip (before biopsy).

A 41-year-old woman was referred to the dermatology clinic of Razi Hospital, Tehran, Iran, with a 6-month history of erosions and crusts on lower lip accompanied by pain and burning sensation (Figure 1). Further physical examination did not reveal any lesion on the skin and mucosa. Multiple topical treatments had been used by the patient in this period but lesions did not improve.

**Figure 2:**
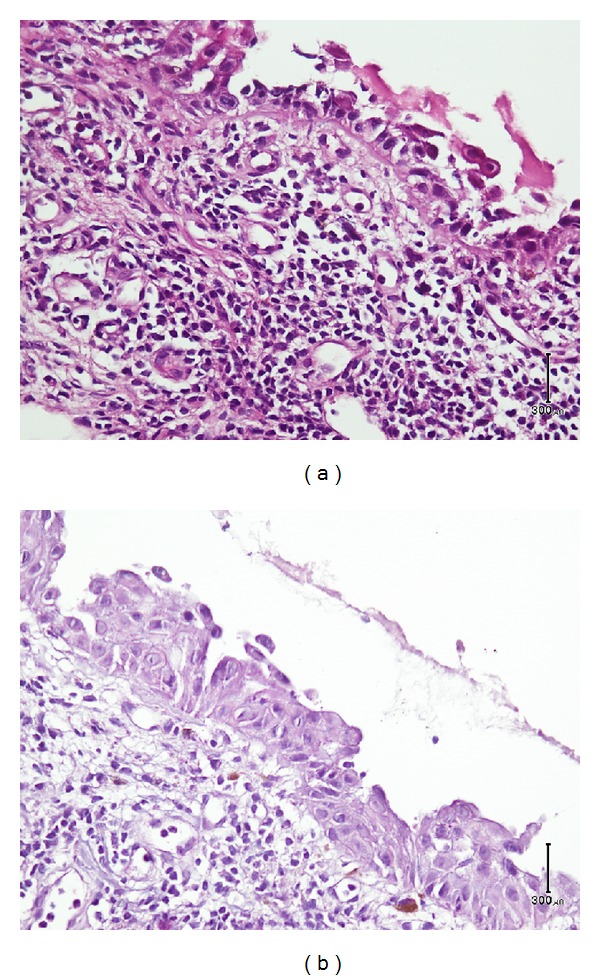
Lip mucosa showing suprabasal acantholysis, clefting, and retraction of tonofilaments.

Our initial differential diagnosis included actinic cheilitis and erosive lichen planus. Biopsy was performed to make definite diagnosis. Histopathological evaluation showed unexpected results of intraepithelial, suprabasal clefting along with keratinocyte acantholysis compatible with pemphigus vulgaris (Figures 2(a) and 2(b)). For the sake of confirmation, we performed rebiopsy and direct immunofluorescence (DIF) studies. DIF study revealed intercellular space deposits of IgG and C3 in the surface epithelium, proving the diagnosis of PV. Quantitative ELISA values of anti-Dsg 1 and anti-Dsg 3 (anti-desmoglein 1 and 3) antibodies were 15 and 56 (positive > 20), respectively.

**Figure 3:**
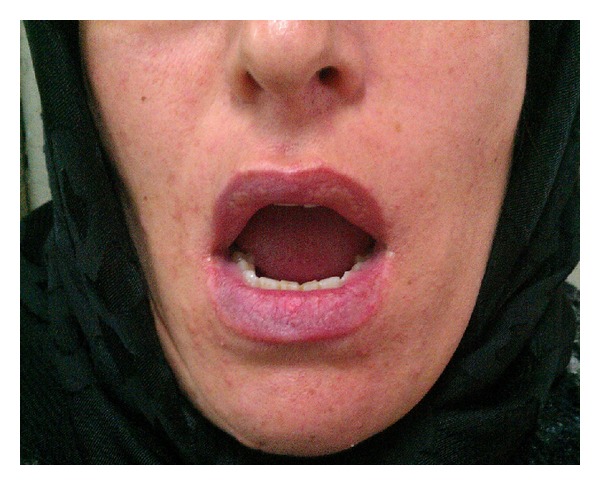
After 2-month follow-up (lesions resolved).

**Figure 4:**
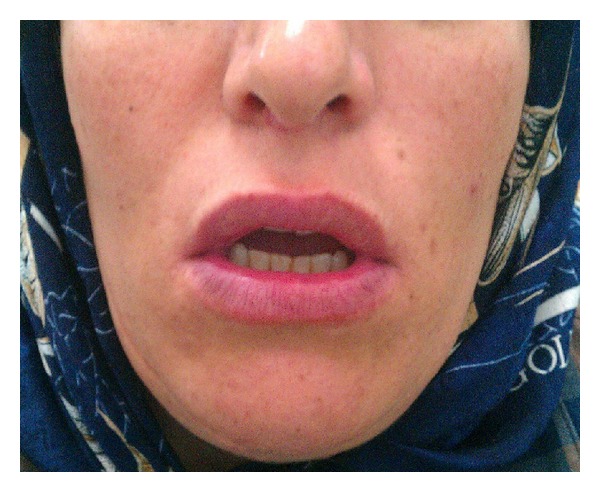
After one-year follow-up.

As the disease was mild and localized, we started cellcept 2 g daily along with 2 sessions of triamcinolone 10 mg/mL intralesional injections after performing initial necessary tests. Lesions were totally resolved within 2 months (Figure 3). After disease remission, treatment continued with cellcept 2 g daily for 1 year follow-up period. There was neither recurrence nor any new lesion elsewhere (Figure 4). Anti-Dsg 1 and anti-Dsg 3 values at the end of 6-month follow-up were 11 and 18.9 (positive >20), respectively.

## 3. Discussion

PV is a chronic autoimmune blistering disease. PV almost always affects the mouth and it can be initial site of presentation in 50% of cases, before skin and other mucosal sites involvement [4]. Diagnosis is based on oral erosions, while confirmation is provided by histological findings, which show the intraepithelial acantholysis. DIF reveals IgG and C3 deposits in intercellular space [5].

In Iran, 62% of PV patients referred to skin clinics had oral lesions [6]. Intact bullae are rarely observed in the oral cavity; in fact, most patients present with irregular erosions with ill-defined borders that tend to heal very slowly and often extend [7]. These erosions are commonly detected in the buccal mucosa and the palate; some cases may progress to involve the pharynx and larynx, causing hoarseness and dysphagia. Other mucous membranes occasionally involved comprise the nasal mucosa, esophagus, conjunctiva, anus, penis, vagina, cervix, and labia [3, 7, 8].

In our patient, disease had some peculiar aspects: (1) the first and only site involved was the lower lip. (2) Disease was mild in such a way that PV was not suspected clinically. (3) Rapid response to intralesional steroid injections without oral steroids and cellcept was used alone in the maintenance phase.

There is only one such case report in the literature, presenting with sole persistent lesion on the lower lip [9] but lesions in our patient were very mild and without hemorrhagic erosions. Interestingly, our patient had positive disease activity shown by high circulating anti-Dsg 3 antibodies. The pathogenesis of pemphigus is thought to be related to the presence of autoantibodies against Dsgs [10–13]. Anti-Dsg antibodies cause disruption to intercellular adhesion in keratinocytes resulting in blister formation [14] but the exact mechanism of how the disease causes such localization in spite of high circulating anti-Dsg antibodies has yet to be determined.

Our patient showed rapid response to intralesional steroid injection and after remission cellcept 2 g daily continued for 1 year and circulating anti-Dsg 3 antibody was in normal range after a 6-month follow-up. Although PV is generally known as a lifetime fatal disease, exceptionally it can be mild and easily managed by steroid sparing agents along with intralesional steroid injection to avoid side effects of systemic corticosteroids.

## 4. Conclusions

This report describes the case of a patient presenting with a 6-month history of persistent crusted lesion on the lower lip, who was finally diagnosed as having PV. Although the main presentation of PV is oral lesions particularly on buccal, palate, and tongue that can extend to gingiva and lips, it is very rare for the disease to present only on the lower lip without involving any other site. We recommend that PV should be taken into account when persistent cheilitis was presented to make early diagnosis. Additionally, we may conclude that localized and solitary lesions of pemphigus vulgaris can be treated and controlled without systemic corticosteroids. Cellcept alone can be used safely and effectively as maintenance therapy in such cases.
